# Serum Protein Gel Agarose Electrophoresis in Captive Tigers

**DOI:** 10.3390/ani10040716

**Published:** 2020-04-20

**Authors:** Daniela Proverbio, Roberta Perego, Luciana Baggiani, Giuliano Ravasio, Daniela Giambellini, Eva Spada

**Affiliations:** 1 Department of Veterinary Medicine (DIMEVET), University of Milan, via dell’Università 6, 26900 Lodi, Italy; luciana.baggiani@unimi.it (L.B.); giuliano.ravasio@unimi.it (G.R.); eva.spada@unimi.it (E.S.); 2 Via Metteotti 9, Almenno San Salvatore, Bergam 24031, Italy; daniela.giambellini@gmail.com

**Keywords:** *Panthera tigris tigris*, *Panthera tigris altaica*, siberian, tigers, bengal tigers, captive, biochemical parameter, serum protein electrophoresis

## Abstract

**Simple Summary:**

The tiger is the largest of the wild cats. There are fewer than 4000 wild tigers (*Panthera tigris*) worldwide and all subspecies of tigers are globally endangered. Due to pressures from poaching and retaliatory killings, this carnivore had lost an estimated 93% of its historic range. Given the critical situation of these wild felines, the health of each individual is of prime importance and laboratory blood testing, as well as evaluation of their physical condition, is important in their health assessment. Protein concentrations in the blood can be altered by malnutrition and dehydration as well as by disease. Serum electrophoresis allows the identification of the different protein fractions present in the blood and represents a useful tool in the diagnosis and monitoring of a number of diseases. Due to the nature of wild *Panthera tigris*, it is extremely difficult to obtain biological samples from free-living subjects, and therefore the values obtained from captive tigers provide very useful data. This study reports serum protein electrophoresis in 11 adult captive individuals. These results will be useful for the evaluation of physiological and pathological alterations in wild and captive tigers and populations.

**Abstract:**

Given the endangered status of tigers (*Panthera tigris*), the health of each individual is important and any data on blood chemistry values can provide valuable information alongside the assessment of physical condition. The nature of tigers in the wild makes it is extremely difficult to obtain biological samples from free-living subjects, therefore the values obtained from captive tigers provide very useful data. Serum protein electrophoresis is a useful tool in the diagnosis and monitoring of a number of diseases. In this study, we evaluated agarose gel serum protein electrophoresis on samples from 11 healthy captive tigers. Serum electrophoresis on all 11 tiger samples successfully separated proteins into albumin, α_1_, α_2_, β_1_, β_2_ and γ globulin fractions as in other mammals. Electrophoretic patterns were comparable in all tigers. Mean± standard deviation or median and range values obtained for each protein fraction in healthy tigers were, respectively: 3.6 ± 0.2, 0.21 (0.2–0.23), 1.2 ± 0.2, 10.7 ± 0.2, 0.4 (0.3–0.6), 1.2 (1–1.8) gr/dL. The results of this preliminary study provide the first data on serum electrophoretic patterns in tigers and may be a useful diagnostic tool in the health assessment of this endangered species.

## 1. Introduction

The tiger is the largest of the wild cats [1]. This large carnivore has lost an estimated 93% of its historic range [2]. Furthermore, across their range, tigers face unrelenting pressures from poaching, retaliatory killings, and habitat loss. Fewer than 4000 wild tigers (*Panthera tigris*) are left in the world [3] and the International Union for Conservation of Nature (IUCN) has classified all subspecies of tigers (*Panthera tigris)* as globally endangered [2].

Approximately 10% of the world’s tigers are found in the Russian Far East, where a single metapopulation represents the vast majority of Siberian, or Amur, tigers (*Panthera tigris altaica*). Today, there are fewer than 400 Amur tigers left in Russia and the eastern region of northeastern China [4,5,6]. The Bengal tiger (*Panthera tigris tigris*) is the most numerous of all tiger subspecies, with a wild population of more than 2500 [7]. 

In parallel, the world’s largest population of tiger lives in captivity. Based on an estimate from a number of conservation organizations, there are approximately 10,000 tigers in captivity all over the world, with as many as 7000 tigers in the US in zoos, sanctuaries or privately owned [8]. Nevertheless, tigers in sanctuaries and zoological gardens worldwide represent a good source of animals for reintroduction into the wild which may play an increasingly important role in preventing the extinction of tigers through captive breeding programs [9]. Furthermore, such facilities are also central in educating the public about the critical status of the endangered tigers throughout the world [10]. 

Given the critical situation of these wild felines, maintaining the health of each individual is essential and any hematological and blood chemistry values that can provide information on the nutritional health status and physical condition are valuable. Furthermore, the detection of signs of disease in these animals is difficult and biological parameters are regarded as very useful complementary tools in the diagnosis and treatment of possible pathologies [11]. Total protein (TP) concentrations and protein fractions can be altered by several factors, such as dehydration, chronic malnutrition, malabsorption, maldigestion, protein-losing enteropathy, severe blood loss, chronic hepatic or renal disease, immunodeficiency, infectious or parasitic disease as well as metabolic or oncologic disorders [12,13]. Electrophoresis enables the separation of serum proteins into four/six fractions (albumin and α_1_, α_2_, β_1_, β_2_ and γ globulin fractions in order of decreasing anodal mobility), resulting in a typical electrophoretic pattern for the distribution of proteins [14]. Serum protein electrophoresis is considered one of the most reliable techniques for determining serum protein composition and, together with a basic hematologic and biochemical profile, are a useful tool in the diagnosis, prognosis, and monitoring of various diseases in both human and veterinary medicine [15]. In veterinary medicine, serum protein electrophoresis is mostly used for the investigation of hypoproteinemia and hyperproteinemia when screening for monoclonal or polyclonal gammopathies [16]. In domestic cats, electrophoretic pattern abnormalities are mainly associated with infectious/inflammatory diseases [17]. Wild cats can be affected by several disorders like infectious agents, including feline immunodeficiency virus (FIV), renal diseases, neoplastic and inflammatory changes [18]. Hypergammaglobulinemia related with myeloma has been reported in wild felid species like tiger [19,20] and lion [21]. Moreover, in captivity, tigers face many stressors and, even if the most effective way to objectively measure stress is by non-invasive measurement of stress hormone levels [9], evaluation of indirect markers of stress may also be useful [22] to ensure holistic wellness and health status [9].The acute phase proteins (APP), migrating in α_1_ and α_2_, globulin fractions, are a group of serum globulins, including ceruloplasmin, haptoglobin, a-2-macroglobulin, alpha1-acid-glycoprotein, that increase during acute inflammation, infection, surgical trauma or stress [22]. It has recently been suggested that phase APP may also be useful in the assessment of animal welfare [23]. 

The wild nature of *Panthera tigris* means it is extremely difficult to obtain biological samples from free-living subjects, making the data derived from captive individuals all the more useful. In addition, access to large numbers of free-living or captive individuals for testing is limited and researchers rely on data from a limited number of individuals [24,25].

To the knowledge of the authors, there is a paucity of literature regarding the serum electrophoretic pattern of *Panthera tigris*. Such data are valuable for the veterinary care of these animals. The objective of this study was to report values of total serum protein and electrophoresis fractions for healthy captive *Panthera tigris*, belonging to the subspecies *Panthera tigris tigris* (Bengala tiger) and *Panthera tigris altaica* (Siberian tiger). These results will be useful for the evaluation of physiological and pathological alterations in wild and captive tiger individuals and populations.

## 2. Materials and Methods 

### 2.1. Animals and Sampling

Sera were collected from fifteen tigers, 6 Bengal tigers (*Panthera tigris tigris*), 7 tigers (*Panthera tigris)* and 2 Siberian tigers (*Panthera tigris Altaica*), including 7 neutered males and 8 neutered females, with ages ranging from 3.5 to 17 years. All tigers were housed, individually or in groups based on individual sociability, in various sized enclosures, rescue centers for exotic felids, zoological parks, or a circus located in northern Italy. Tigers were being immobilized for routine physical examination, ocular and dental examination, vaccination administration or minor surgical or diagnostic procedures. Each tiger received a clinical examination, and those with any visible abnormalities, with an inadequate body condition score, with signs of dehydration or with any signs of disease were excluded from the group of subjects considered clinically healthy. 

### 2.2. Sample Preparation

As part of the health examination while under general anesthesia, blood samples were collected from the jugular, cephalic or saphenous vein of each animal. Blood was collected in plain tubes (Sistema BD Vacutainer^®^, Becton Dickinson Italia SPA, Italy) and serum was obtained by centrifugation at 10 min at 2500× *g*. Written owner consent for use of surplus blood samples, and use of data for scientific purposes, was obtained during consultations. The study design was approved by University of Milan Animal Welfare Bioethical Committee (Approval number OPBA 31/2019). 

### 2.3. Total Serum Protein 

Total serum protein concentration was measured by spectrophotometry using the colorimetric biuret method (Hagen Diagnostica S.R.L., Via Pratese 13 Firenze) on a Cobas Mira Classics Roche automated chemistry analyzer (Roche S.p.A., Mannheim, Germany).

### 2.4. Agarose Gel Electrophoresis

Sera samples were refrigerated at 4 °C and were analyzed within 8 hours of sampling. Protein fractions were analyzed using a semiautomated agarose gel electrophoresis (AGE) system (with HYDRAGEL Kit β_1_-β_2_ (SEBIA, Issy-les-Moulineaux, France). Serum was electrophoresed for 7 minutes at 33 volts hours and stained with diluted Amidoschwarz dye at pH 2 (4 g/L Amidoschwarz dye and 6.7% ethylene glycol). The AGE procedure was conducted according to the manufacturer’s instructions, and commercial human serum was used as the control (normal control serum, Sebia, Evry, France). Using the computer software Phoresis for Windows 2000 or XP Pro (Sebia), the electrophoretic curve for each sample was displayed. Protein fractions were determined as the percentage optical absorbance, and the absolute concentration in g/dL was automatically calculated from the total serum protein concentration. Albumin to globulin (A/G) ratios were also calculated. The same operator analyzed all samples. To establish the inter-assay-accuracy of agarose gel electrophoresis on tiger serum, sera from two healthy tigers, one Bengal Tiger, 9A, F and one Siberian Tiger 1,1A, F, were tested 3 times on the same day, in the same laboratory and interpreted in duplicate by two operators. Coefficient of variability (CV) computed as SD/mean × 100 was calculated for each protein fraction.

### 2.5. Statistical Analysis 

Data were tested for normality using the Shapiro–Wilk normality test. For normal distributions, means and standard deviations were calculated, and for non-normally distributed data, medians and ranges were calculated. Due to the scarcity of information on tiger electrophoretic patterns, values were compared to cheetah [13] and domestic cat [17] reference values. Due to the small sample size, the reference interval (RI) limits are directly estimated by the minimum and maximum values [26]. Statistical analysis was performed using MedCalc Statistical Software version 15.11.3 (MedCalc Software, Ostend 8400, Belgium). 

## 3. Results

**Figure 1:**
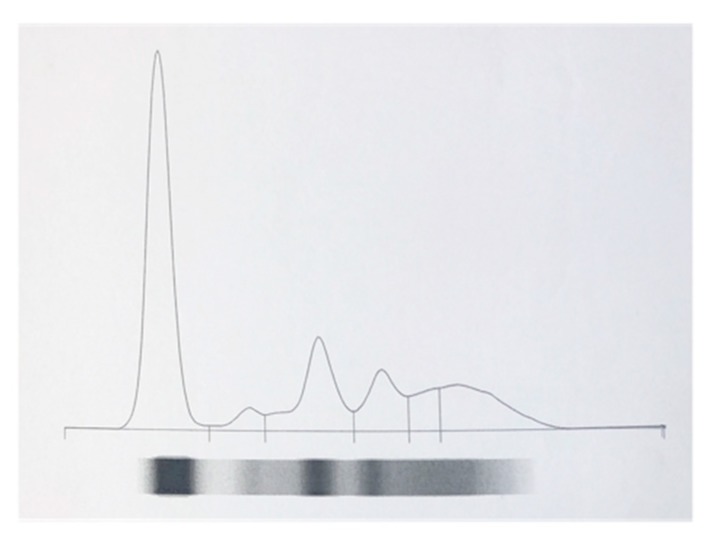
Electrophoretic curve of one healthy tiger. Agarose gel electrophoresis was able to separate serum proteins into six fractions: albumin, α1, α2, β1, β2 and γ globulin in order of decreasing anodal mobility.

After clinical examination, only 11 out of 15 tigers were deemed clinically healthy. Therefore, serum samples from 11 tigers—five Bengal tigers (*Panthera tigris tigris*), five tigers (*Panthera tigris)* and one Siberian tiger (*Panthera tigris Altaica*), four neutered males and seven neutered females, with ages ranging from 5 to 16 years—were used to identify electrophoretic patterns of serum proteins in healthy tigers. Agarose gel electrophoresis carried out on all 11 tiger samples, successfully separated tiger serum proteins into albumin, α_1_, α_2_, β_1_, β_2_ and γ globulin fractions (Figure 1). All tigers had comparable electrophoretic patterns. 

All data, with the exception of α_1_, β_2_ and γ globulin fractions, were normally distributed. Gender-specific and age differences were not analyzed because the sample size was insufficient to allow statistical evaluation. Descriptive statistics of the protein serum electrophoresis fractions carried out in our healthy tiger population and reference values for cheetahs [13] and domestic cats [17] are given in Table 1. 

The mean values of α_2_ and β_1_ globulin were 11.65% and 55.55% respectively, above the higher reference values indicated for domestic cats for the same globulin fractions [17]. Mean values of α_1_, α_2_ and γ globulin fractions were, respectively, 13.04% below the lower value and 74.24% and 18.18% above the higher value references indicated for cheetahs for the same globulin fractions [13]. The inter-assay accuracy of the agar gel electrophoresis in tiger serum was excellent as the same electrophoretic shape was recorded in all three repeated samples and for all protein fractions, with the exception of the α_1_ globulin fraction in tiger number 2, the CVs were within the accepted ranges of within-subject biological variation for people (Table 2) [27].

## 4. Discussion

To the best of our knowledge, there have been no studies of the serum protein electrophoretic fractions in healthy tigers. The total serum protein electrophoretic pattern obtained with agarose gel electrophoresis separated the protein into six fractions, albumin, α_1_, α_2_, β_1_, β_2_ and γ globulins as in other mammals [14], resulting in a typical electrophoretic pattern for the distribution of proteins. Mean values of albumin and globulin fractions, with the exception of α_2_ and β_1_ globulins mean concentration, in our healthy tiger population fell within the reference values for domestic cats [17]. The average values of α_2_ and β_1_ globulins were above the higher reference values indicated for protein serum electrophoresis performed with AGE in domestic cats [17]. In mammals, the α_2_ globulin fraction mainly consists of acute-phase proteins, such as α_1-_ acid glycoprotein, and often these proteins increase as a result of activation of the inflammatory response [28] to regulate different stages of inflammation [29]. Complement is one of the main proteins present in the β globulin fraction, corresponding to the sum of β_1_ and β_2_ globulin fractions [14]. Both α_2_ and β_1_ globulins fractions may be elevated if there is increased production of some acute phase proteins which migrate into these regions [14]. Recently, acute phase proteins have also been proposed as useful stress biomarkers. In humans, cows and experimental animals, psychological and physical stress elevates plasma interleukin-6 and APP levels [22,30]. Acute phase proteins are synthesized predominantly in the liver, in response to secretion of pro-inflammatory cytokines. In response to stress signals, the hypothalamic–pituitary–adrenal (HPA) axis may trigger cytokine production resulting in an increase in hepatic APP synthesis and release into the bloodstream [22]. Although lacking specificity, the detection of an increase in α_2_ globulin could help in monitoring the stress status of tigers in captivity. 

Although the sample size analyzed was limited, the data obtained in this study could suggest that healthy tigers may have a higher concentration of acute phase proteins than domestic cats, or that the tiger population studied could have been in an inflammatory state. The interpretation of serum protein electrophoretic patterns depends on the variations among different groups of animals. Moreover, acute phase proteins are a variable group of serum proteins and concentrations vary widely between different animal species [31]. Domestic cats are in the same family and share a similar physiology to tigers and could be an acceptable alternative for comparison of normal values for many biochemical parameters; however, they are a different subfamily and extrapolation of all results is dangerous. Depauw et al. (2014) [13] reported results of captive cheetah (*Acinonyx jubatus*) serum protein electrophoretic fractions by capillary electrophoresis (CE). Although the AGE and CE are different techniques for protein fraction separation, the shape of the electrophoretogram of cheetah serum was comparable to that found in our healthy tiger sample [13].

The percentage of variation observed between protein fractions of two healthy tiger serum samples repeatedly submitted to agarose gel electrophoresis to evaluate inter-assay-accuracy were within the accepted ranges of within-subject biological variation for people [27]. Only the α_1_ globulin fraction exceeded the acceptable value of 20.83%. This result could be due to the low concentration of α_1_ globulins in the serum. In fact, the accuracy of analysis is usually better for protein fractions found in higher serum concentrations because low concentrations are more susceptible to small changes [32]. Cushing et al. (2019) [19] described a cases series of myeloma associated with hypergammaglobulinemia in five adult tigers. Diagnosis of myeloma is based on a variety of clinical signs often associated with monoclonal gammopathy found in serum. It is interesting to note that in this case series, the serum protein electrophoresis was done in the absence of reference values for the serum protein pattern typical of this species. This underlines the importance of acquiring a database of reference values even for the rarest wild carnivores.

There were a number of limitations with this study. Firstly, although each tiger was clinically examined, and screened for visible alterations and low body condition score, the history was sometimes incomplete or unavailable, which may have compromised the accurate categorization of animals according to health status and disease type. In addition, samples were from animals in different types of housing, so the diversity of habitat and diet could have affected the results. [25]. Furthermore, due to the small number of subjects, we are not able to define reference ranges. In fact, following the reference interval guidelines of the American Society for Veterinary Clinical Pathology, reference ranges should not be calculated when the sample size is <20 subjects. In these cases, mean or median and minimum and maximum values should be provided [33]. For the same reason, we did not evaluate the influence of gender or age on serum protein electrophoretic patterns. 

In veterinary medicine, serum protein electrophoresis is recognized as a useful tool in the diagnosis, prognosis and monitoring of a number of diseases [15,17]. Alterations of blood biochemical values are interpreted by comparison with the same value in healthy subjects. This study provides useful data on the serum protein electrophoretic values in healthy captive tigers that increases our understanding of this endangered species. Furthermore, the paucity of reports of variations in serum protein electrophoretic patterns in free-ranging or captive tigers makes this preliminary study a useful aid for the evaluation of physiological and pathological alterations in both wild and captive tiger populations. 

## 5. Conclusions

Serum protein electrophoresis is a useful tool in the diagnosis and monitoring of a number of diseases. This study presents serum protein electrophoresis in a sample of healthy captive tigers. Due to the nature of wild *Panthera tigris*, it is extremely difficult to obtain biological samples from free-living subjects, and therefore the values obtained from captive tigers provide very useful data. Results indicate that agarose gel electrophoresis separates the total serum protein into six fractions, albumin, α_1_, α_2_, β_1_, β_2_ and γ globulins in tigers as in other mammals, resulting in a typical electrophoretic pattern for the distribution of proteins. Mean values of albumin and globulin fractions, with the exception of α_2_ and β_1_ globulins mean concentration, in our healthy tiger population fell within the reference values indicated for protein serum electrophoresis performed with AGE in domestic cats. These preliminary results provide the first data on serum electrophoretic pattern in healthy tigers and may offer a platform for further research into serum proteins as a useful diagnostic tool in the health assessment of this endangered species. 

## Figures and Tables

**Table 1:** Total protein concentration and concentrations of albumin and α1, α2, β1, β2 and γ globulin fractions, obtained using agarose gel electrophoresis (AGE), in 11 healthy captive tigers. Mean, SD, minimum and maximum value. Reference value of electrophoretic fractions in cheetah and domestic cats from previous studies. ° data non-normally distributed.

Parameter	Mean +/− SD (range)	Minimum Value	Maximum Value	Reference Values Cheetah Depauw (2014)	Reference Values Domestic Cat Taylor (2010)
TP g/dL	7.4 ± 0.8	6.2	8.9		
Albumin g/dL	3.6 ± 0.2	3.3	3.9	3.2–4.8	2.9–4.67
α_1_ globulin° g/dL	0.2 (0.2–0.23)	0.2	0.26	0.23–0.67	0.20–0.49
α_2_ globulin g/dL	1.2 ± 0.2	0.8	1.5	0.13–0.66	0.29–1.03
β_1_globulin g/dL	0.7 ± 0.2	0.44	1.2	0.4–0.8	0.15–0.45
β_2_ globulin° g/dL	0.4 (0.3–0.6)	0.3	0.6	0.16–0.48	0.15–0.49
γ globulin° g/dL	1.2 (1–1.8)	0.8	2.3	0.29–1.1	0.43–2.14
A/G	0.92 ± 0.2	0.7	1.3	1.6	

**Table 2:** Serum protein electrophoresis in 2 healthy captive tigers serum samples tested 3 times on the same day, in the same laboratory and interpreted in duplicate by two operators. Coefficient of variability (CV) of total protein, albumin and α_1_, α_2_, β_1_, β_2_ and γ globulin calculated as SD/mean × 100.

Tiger	Total Protein g/dL	Albumin g/dL	α_1_globulin g/dL	α_2_globulin g/dL	β_1_globulin g/dL	β_2_globulin g/dL	γglobulin g/dL	A/G
Tiger 1	7	3.32	0.22	1.16	0.62	0.43	1.25	0.9
Tiger 1	7	3.19	0.23	1.23	0.62	0.41	1.33	0.84
Tiger 1	7	3.3	0.22	1.19	0.58	0.43	1.27	0.89
CV	0	2.14	2.27	2.94	3.83	2.61	3.2	4,8
Tiger 2	8.9	3.67	0.2	1.5	0.69	0.56	2.29	0.7
Tiger 2	8.9	3.89	0.15	1.41	0.66	0.5	2.3	0.78
Tiger 2	8.9	3.7	0.17	1.49	0.68	0.6	2.27	0.71
CV	0	3.2	14.4	3.4	1.7	9.1	0.6	5.4
CV_1_		3.2	11.4	10.3	10.1	−	14.6	

CV_1_: within-subject biologic variation for human samples (Westgard https://www.westgard.com/biodatabase1.htm).
